# Supplementary material for: Exploring applications of crowdsourcing to cryo-EM
Source: J Struct Biol. Author manuscript; Available in PMC 2018 Aug 10. (PMC6086358; doi:10.1016/j.jsb.2018.02.006)
Supplement: 1 [file NIHMS976652-supplement-1.zip › tableS1.pdf]

Dataset	Number of Images	Classifications per Image	Total Number of Classific
Inititial User Testing #1	200	5	
Inititial User Testing #2	200	5	
Inititial User Testing #3	200	5	
HIV Trimer	20	5	
TRPV2 Ion Channel	20	5	
hline			
