# Supplementary figures and images for: Exploring applications of crowdsourcing to cryo-EM
Source: J Struct Biol. Author manuscript; Available in PMC 2018 Aug 10. (PMC6086358; doi:10.1016/j.jsb.2018.02.006)

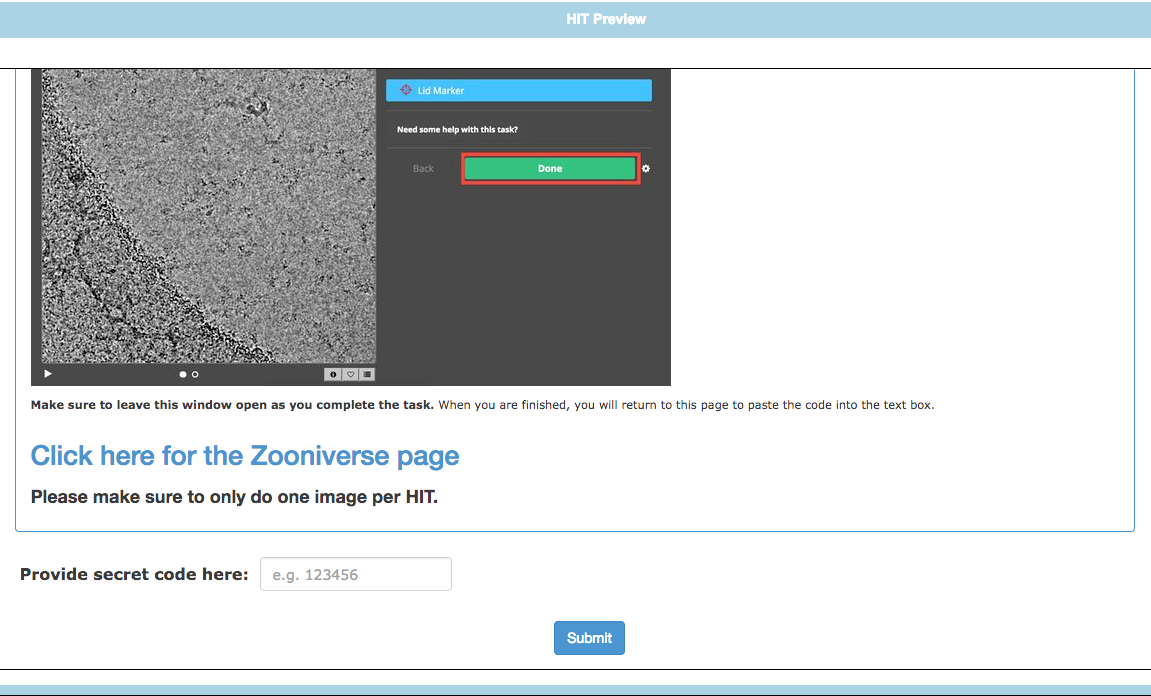

Supplement: 3 [file NIHMS976652-supplement-3.tif]

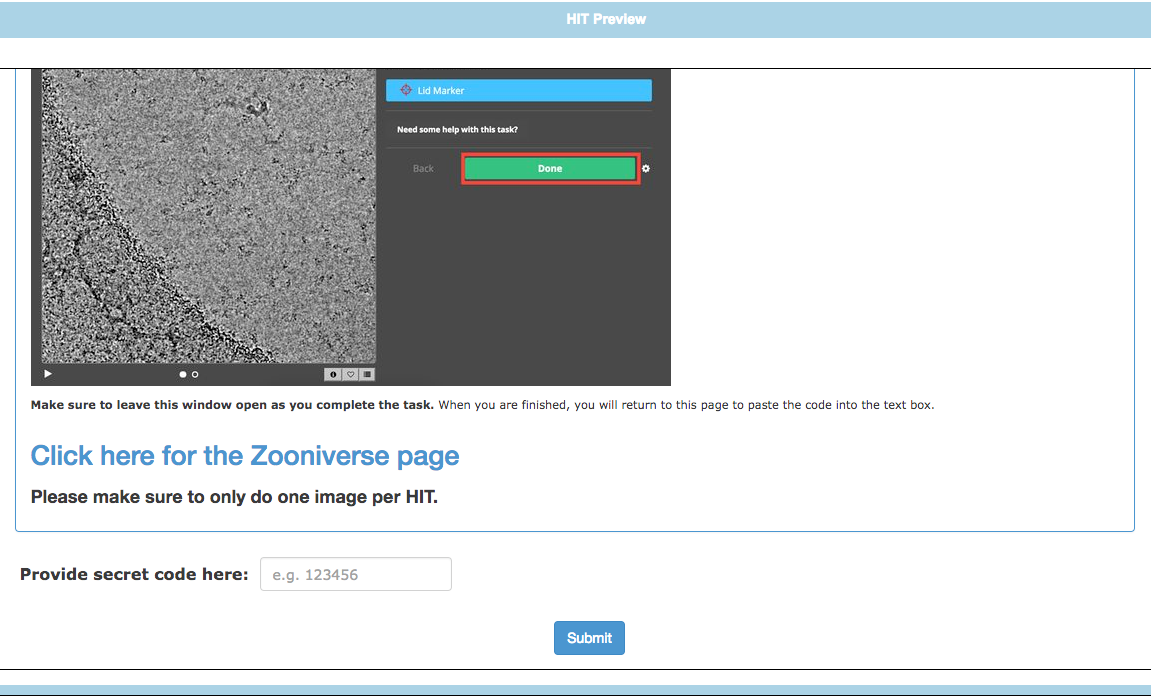

Supplement: 4 [file NIHMS976652-supplement-4.tif]

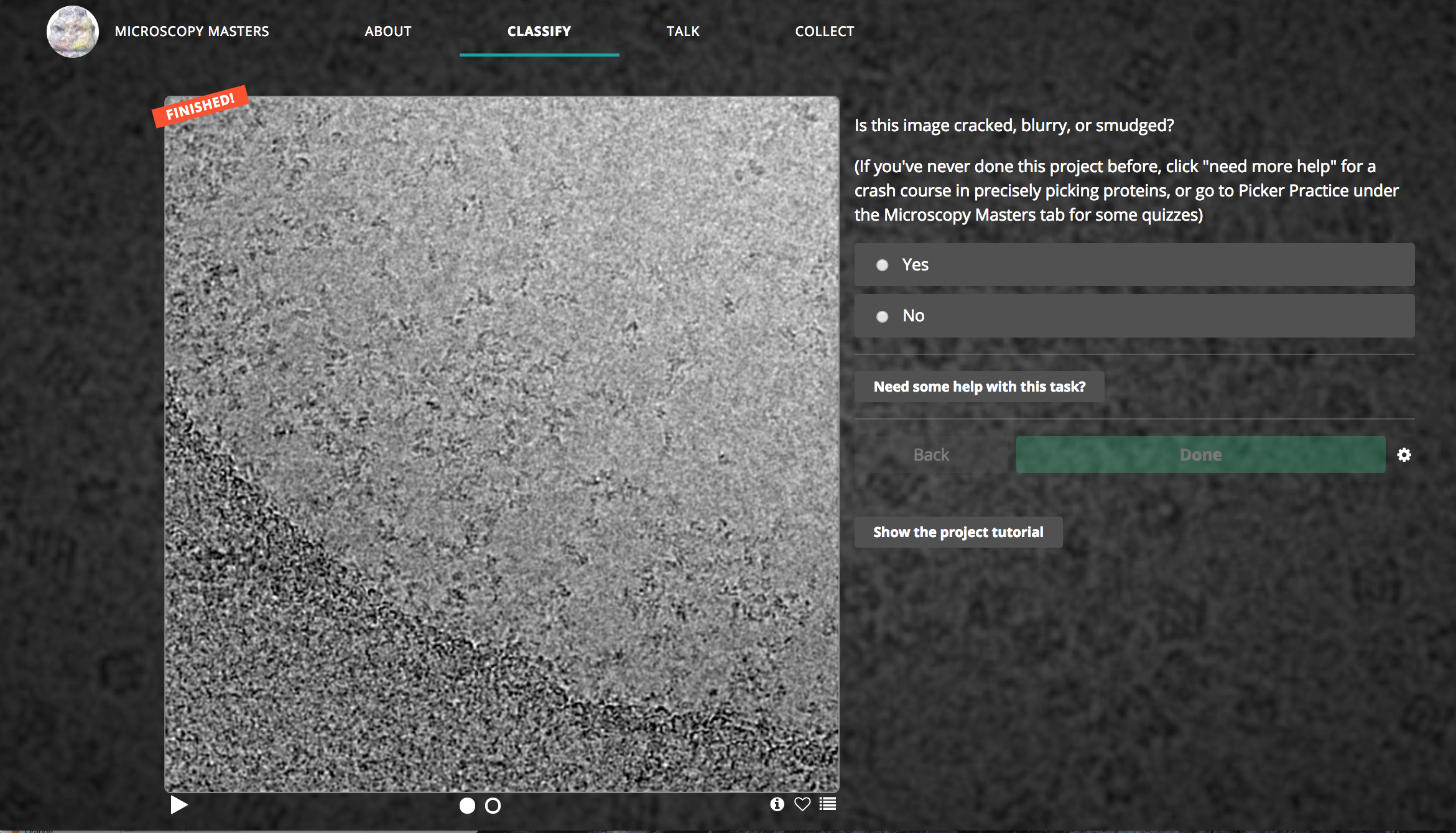

Supplement: 5 [file NIHMS976652-supplement-5.tif]

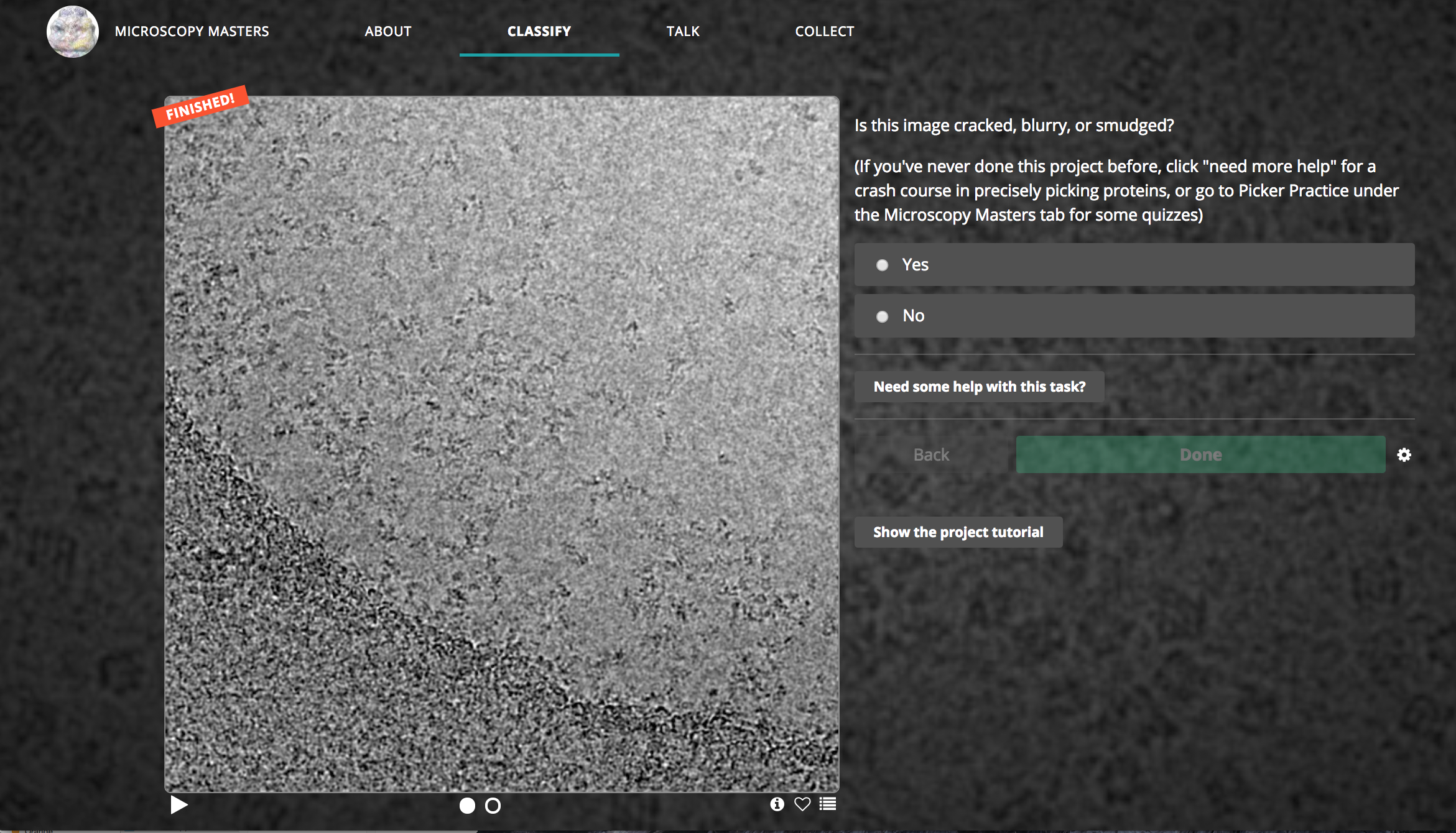

Supplement: 6 [file NIHMS976652-supplement-6.tif]

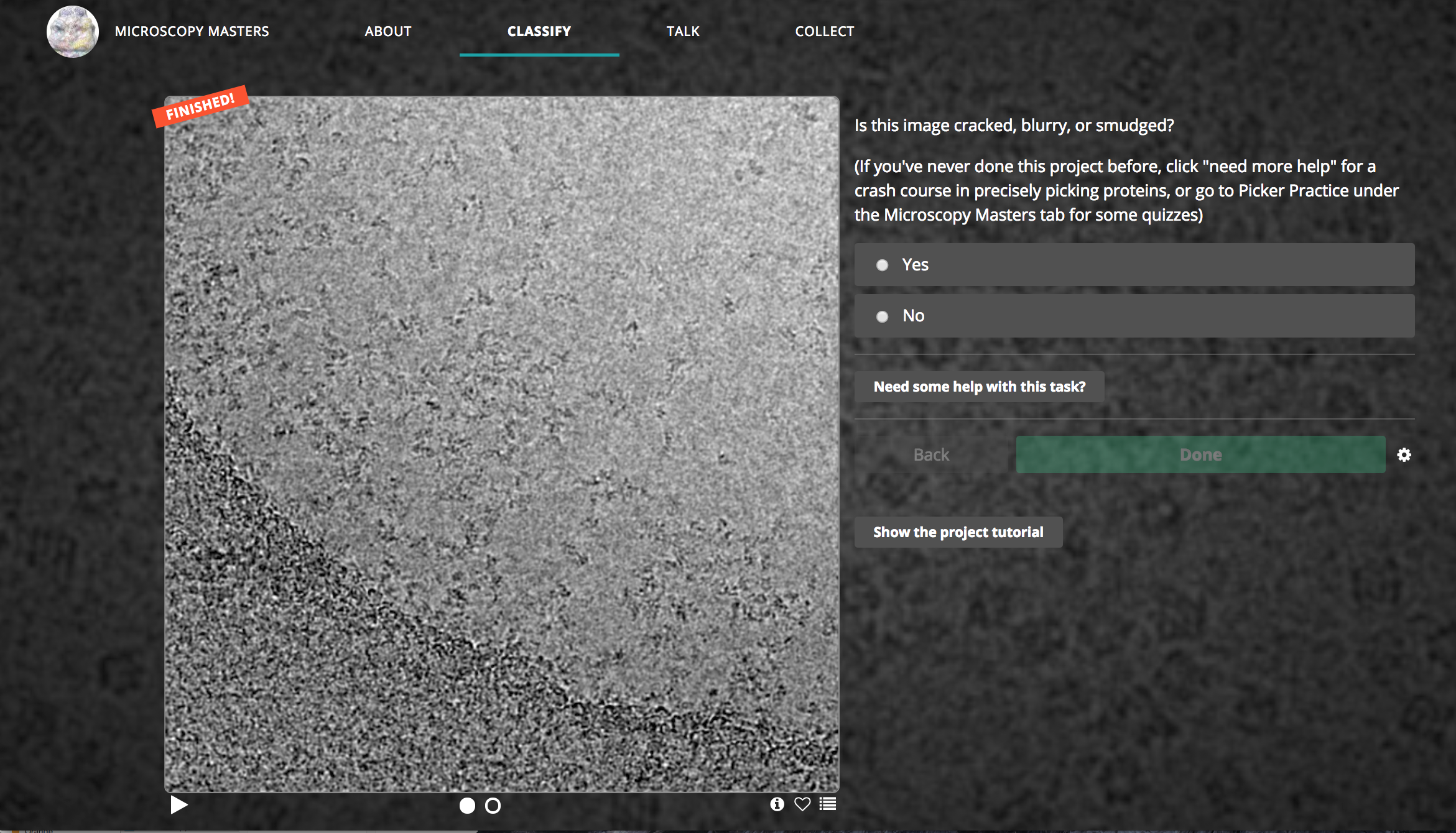

Supplement: 7 [file NIHMS976652-supplement-7.tif]

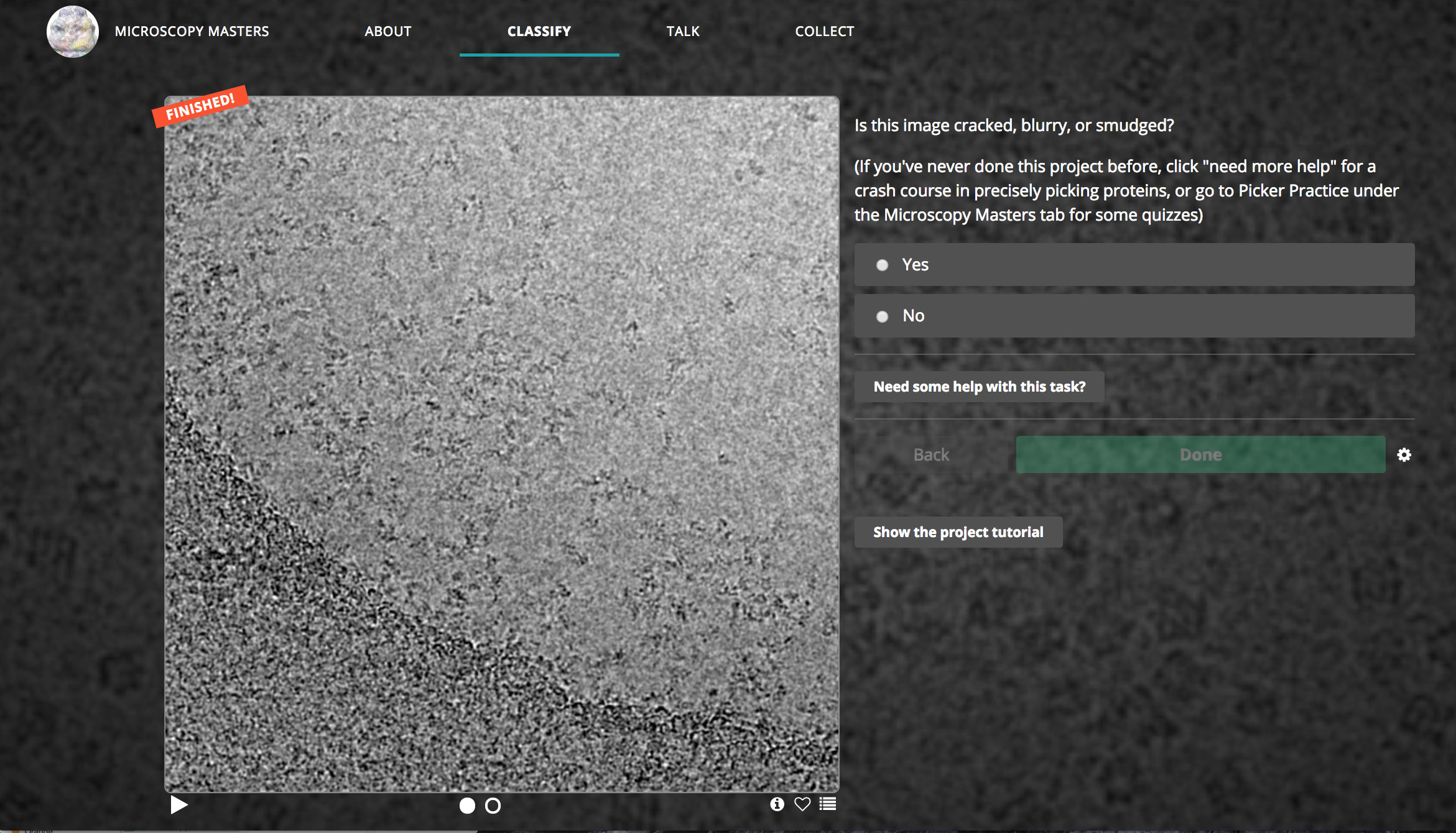

Supplement: 8 [file NIHMS976652-supplement-8.tif]

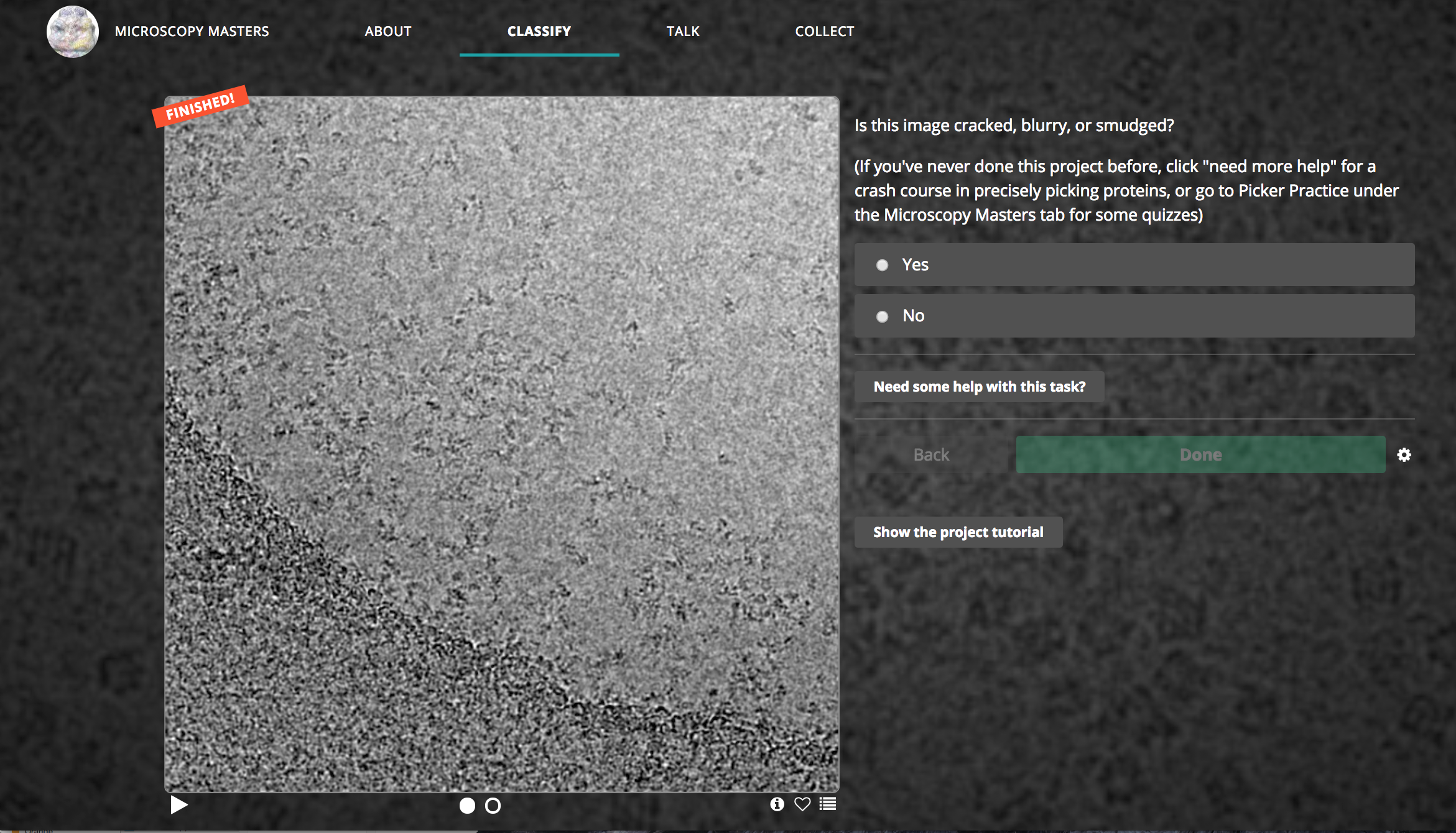

Supplement: 9 [file NIHMS976652-supplement-9.tif]
